# Dual release of daptomycin and BMP-2 from a composite of β-TCP ceramic and ADA gelatin

**DOI:** 10.1186/s12896-024-00863-4

**Published:** 2024-06-03

**Authors:** Lucas Ritschl, Pia Schilling, Annette Wittmer, Annerose Serr, Hagen Schmal, Michael Seidenstuecker

**Affiliations:** 1 G.E.R.N. Tissue Replacement, Regeneration & Neogenesis, Department of Orthopedics and Trauma Surgery, Medical Center- Albert-Ludwigs-University of Freiburg, Faculty of Medicine, Albert-Ludwigs-University of Freiburg, Hugstetter Straße 55, 79106 Freiburg, Germany; 2 Institute of Microbiology and Hygiene, Faculty of Medicine, Medical Center Albert-Ludwigs-University of Freiburg, Hermann- Herder-Straße 11, 79104 Freiburg, Germany; 3 Department of Orthopedics and Trauma Surgery, Medical Center-Albert-Ludwigs-University of Freiburg, Faculty of Medicine, Albert-Ludwigs-University of Freiburg, Hugstetter Straße 55, 79106 Freiburg, Germany

**Keywords:** Dual release, Daptomycin, BMP-2, β-TCP scaffold, ADA-gelatin gel, Bone infection

## Abstract

**Background:**

Antibiotic-containing carrier systems are one option that offers the advantage of releasing active ingredients over a longer period of time. In vitro sustained drug release from a carrier system consisting of microporous β-TCP ceramic and alginate has been reported in previous works. Alginate dialdehyde (ADA) gelatin gel showed both better mechanical properties when loaded into a β-TCP ceramic and higher biodegradability than pure alginate.

**Methods:**

Dual release of daptomycin and BMP-2 was measured on days 1, 2, 3, 6, 9, 14, 21, and 28 by HPLC and ELISA. After release, the microbial efficacy of the daptomycin was verified and the biocompatibility of the composite was tested in cell culture.

**Results:**

Daptomycin and the model compound FITC protein A (*n* = 30) were released from the composite over 28 days. A Daptomycin release above the minimum inhibitory concentration (MIC) by day 9 and a burst release of 71.7 ± 5.9% were observed in the loaded ceramics. Low concentrations of BMP-2 were released from the loaded ceramics over 28 days.

## Introduction

Osteomyelitis is an inflammation that typically involves the bone (osteitis), bone marrow (osteomyelitis), and periosteum (periostitis) [1]. The adjacent soft tissue may also be impacted. This condition is instigated by various microorganisms, resulting in bone destruction [2]. Etiologically, osteomyelitis is categorized as hematogenous, locally transmitted (per continuitatem), exogenous, and specific [3]. In pediatric cases, hematogenous osteomyelitis predominantly affects long bones, while in adults, it often presents as spondylitis [4]. Infections can originate from sources such as common skin infections or contamination during intravenous drug administration, leading to hematogenous dissemination into the bone and subsequent osteomyelitis [5]. *Staphylococcus aureus (S. aureus)* is the primary causative agent for primary hematogenous and locally transmitted osteomyelitis [5]. Notably, in implant-associated infections, small colony variants (SCV) of Staphylococcus and coagulase-negative Staphylococcus should be considered [6]. Osteomyelitis can be initially classified into acute and chronic forms based on the infection duration or histological inflammation type [7]. Chronic osteomyelitis is identified when the causative agent persists for more than 6 weeks, although the distinction may be nuanced [3]. Conversely, the presence of sequestra on CT or MRI serves as a definitive criterion for chronic infection [4]. Sequestra refers to necrotic bone fragments rejected by healthy tissue, hindering infection resolution [8]. The usual therapy consists of debridement, i.e. surgical removal of the infected tissue, and systemic antibiotic therapy [9] tailored to the specific pathogen. Local antibiotic therapy is much more effective [10] and is sometimes already applied with the use of gentamicin-loaded PMMA chains (Septopal®) [11]. The disadvantage, however, is that they have to be removed again in a second surgical procedure and, in addition, a not inconsiderable proportion of the antibiotics remains in the PMMA [12] because it is biodegradable. Various processes have been described for loading porous ceramics with additives such as antibiotics and pharmaceuticals. The manner in which these agents are loaded is especially critical for their release. One can spray the active substances onto the surface. In addition, regardless of the manufacturing process, it is possible to incubate the ceramics for a certain time in an aqueous solution containing antibiotics [13–18]. . Droplet loading, in which the ceramic is not immersed in a solution but the active ingredient solution is applied to the ceramic via a drip process, is another option [19]. These procedures have in common that a subsequent drying phase is necessary, this can include several procedures: in an oven with a temperature of max. 50 °C to avoid the destruction of the antibiotics [15, 16, 20], both air and vacuum drying [21–24] are described in the literature. As the load is adhesive, these methods are usually applied for short-term drug release [25]. To prolong the release, the active ingredients can be encapsulated, as is done in the pharmaceutical industry in the production of tablets and capsules. For this purpose, a layer is applied as a diffusion barrier after the loading process. This can be done by dipping, dropping or spraying [26]. The literature has previously documented a delayed in vitro drug release from a carrier system [27], comprising microporous β-TCP ceramic and alginate [28–31]. Comparatively, ADA gelatin gel has exhibited superior mechanical properties when incorporated into β-TCP ceramic and demonstrated higher biodegradability than pure alginate [32]. Given its prior assessment for drug release [31, 33], ADA gelatin gel emerges as a promising hydrogel for targeted and controlled drug delivery. Daptomycin, a relatively new antibiotic in the gram-positive spectrum, and BMP-2, a substance that promotes bone healing, are of interest for local application. Notably, the potential for dual drug release in this system remains unexplored, representing an avenue for enhancing patient outcomes by combining anti-infective treatment, bone growth promotion therapy, and improved bone stability facilitated by the β-TCP ceramic.

## Materials and methods

### Preparing the microporous β-TCP ceramics

The described ceramics were manufactured according to the previously outlined procedure [34–37]. A mixture of 80 g α-tricalcium phosphate and 20 g tricalcium phosphate (Art. No. 102143, Merck, Darmstadt, Germany; a blend of apatite and calcium hydrogenphosphate) was combined with 60.0 ± 0.2 g of a solution containing 0.2 M Na2HPO4 (Art. No. S9763, > 99%, Sigma Aldrich, St. Louis, USA) and 1% polyacrylic acid (Art. No. 81132, Fluka, Hannover, Germany; Mw = 5.1 kDa). The mixture was stirred at 2000 rpm using a four-wing impeller for 45s (Eurostar Digital, IKA, Staufen, Germany), and the resulting paste was poured into plastic syringes after removing their tips (Ø = 23 mm). After 45 min of hardening, the paste was covered with 10 mL of PBS 7.4 solution (Art. No. P5368, Sigma, St. Louis, USA) and incubated at 60 °C for 3 days. The samples (Ø = 23 mm; L = 70 mm) were then dried at the same temperature and sintered at 1250 °C for 4 h, with heating and cooling occurring at a rate of 1 °C per minute. The cylinders were subsequently trimmed to a length of 26 mm and a diameter of 7 mm. Finally, the ceramics were washed overnight in ethanol (Art. No., 98%, Fluka, Hannover, Germany) to eliminate residual particles and calcined at 900 °C to eliminate all organic residues. Before usage, the ceramics were shortened to cylinders with a length of 6 mm and washed again. The samples underwent sterilization in a drying oven (Memmert UN55, Memmert, Schwabach, Germany) at 200 °C for 4 h.

### Characterization of the microporous β-TCP ceramics

The characterization procedures closely mirrored those detailed in prior publications [30, 38]. The structural and pore size analyses were conducted using Environmental Scanning Electron Microscopy (ESEM) (FEI Quanta 250 FEG, Hillsboro, USA), Micro-computed Tomography (µCT) (Scanco Micro-CT 50, Brüttisellen, Switzerland), and porosimetry (Porotec Pascal 140/440, Hofheim, Germany). ESEM operated at an accelerating voltage of 10 kV, while µCT parameters were set at 90 kV, 4 W, 44 µA, with a resolution of 2 μm and an integration time of 5000 ms. Porosimetry, performed using the Pascal 140 instrument, covered pore sizes ranging from 1000 μm to 1.4 μm, with a pressure increase to 0.1 kPa. The Pascal 440 porosimeter addressed pore sizes from 1.4 μm to 1.8 nm, with a pressure increase to 400 MPa. To determine the composition of the ceramics, Energy-Dispersive X-ray Spectroscopy (EDX) was employed (Oxford Instruments, Abingdon, UK), with an accelerating voltage of 12 kV and a measurement time of 100 s (live time-corrected). X-ray Diffraction (XRD) analysis was carried out using Bragg-Brentano geometry equipped with a Cu anode, secondary graphite monochromator, scintillation counter, 40 kV/40 mA, 1°2-theta/min, and a step size of 0.02°2theta.

### Preparation and characterization of the ADA-gelatin hydrogels

Gelatin and alginate were sterilized by using low temperature hydrogen peroxide gas plasma sterilization (pressure 63.3 Pa; temperature 50 °C; diffusion time 8 min; plasma time 4 min; H_2_O_2_ concentration 6 wt%; peroxyacetic acid 1 wt%) [39]. The fabrication of alginate di-aldehyde (ADA) was carried out as already described by us elsewhere [38]. Daptomycin hydrochloride (Cubicin 500 mg Daptomycin i.v.; MSD Sharp&Dohme; PZN 06708869) was dissolved together with gelatin in a final concentration after mixing ADA and gelatin 1:1 together of 50 mg/mL. 1.5 g ADA from plasma-sterilized alginate were dissolved in 600 µl BMP stock solution (equivalent to 300 µg BMP-2) and 29.4 mL PBS.

#### Gel permeation chromatography (GPC)

GPC analysis was conducted to ascertain the molar mass distribution and mean molar masses of the employed alginate, ADA, and gelatin. A 20 mg sample was dissolved in 10 mL of the eluent over two days at room temperature. Prior to measurement, the solutions underwent filtration through a PTFE filter membrane with a porosity of 1 μm. Calibration involved the use of various pullulan standards in the separation area of the column combination (PSS Suprema 10 μm pre-column, ID 8.0 mm × 50 mm; PSS Suprema 10 μm, 100 Å, ID 8.0 mm × 300 mm; PSS Suprema 10 μm, 3000 Å, ID 8.0 mm × 300 mm; PSS Suprema 10 μm, 3000 Å, ID 8.0 mm × 300 mm (PSS, Mainz, Germany)). As eluent, 0.02 M phosphate buffer at pH of 6.6 and 0.5 M NaCl aq. was used.

#### Rheology

Rheological investigations were conducted using the Malvern Kinexus lab + KNX2110 rheometer (Malvern, UK). The cone plate (CP1/40 SR3033 SS) employed had a diameter of 40 mm and an angle of 1°, with a distance to the fixed plate (PLS40 S2345 SS) set at 23 μm. The measurements, employing a frequency ramp, were carried out with a shear strain of 1% and at a temperature of 25 °C within the range of 0.02 Hz to 16 Hz. The measurements were performed for ADA and gelatin gels separately, as well as for ADA-gelatin (with/without daptomycin and BMP-2). 1 mL of gel was applied to the plate. After moving the plates together until the gap width was reached, any gel that escaped laterally was wiped off with cellulose.

### Loading the microporous β-TCP ceramics

The loading principle developed by Seidenstuecker [28] using a flow chamber was used and 6 chambers were connected in parallel to the vacuum pump (KNF Neuberger SC920, Freiburg, Germany) so that several ceramics could be loaded at the same time. After the loading process, the ceramics were placed in 30 mM CaCl_2_ (Sigma Aldrich, St. Louis, USA) solution for crosslinking. The CaCl_2_ solutions also contained the same concentration of daptomycin and/or BMP-2 as in the gel. The distribution of the gel in the ceramic has already been measured in a previous work [30, 38] using fluorescein and fluorescence microscopy. Therefore, we have not done so here.

### Release experiments

The ceramics containing the load were reweighed and transferred to 5 mL vials following the same procedure as the unloaded negative controls. They were then covered with 3 mL of distilled water and positioned in a warming cabinet, shielded from light, at 37 °C for a duration of 28 days. On days 1, 2, 3, 6, 9, 14, 21, and 28 post-experiment, the ceramics were relocated to a new 5 mL vial containing fresh distilled water, while the previous vial was frozen at − 20 °C.

### Determination of the release kinetics of daptomycin and BMP-2

Each sample was thawed and subjected to sterile filtration using 0.2 μm disposable filters (Chromafil Xtra H-PTFE-20/25, Art. No. 729245, Macherey-Nagel, Düren, Germany). The antibiotic concentration in the samples was assessed through High-Performance Liquid Chromatography (HPLC), while the BMP2 content was determined using Enzyme-Linked Immunosorbent Assay (ELISA).

#### HPLC

Daptomycin determination using High-Performance Liquid Chromatography (HPLC) was performed with the Shimadzu CBM-20 A, CTO-20AC, DGU-20A5R, LC-20ADXR, Reservoir Tray, RF-20 A, SIL-30AC, SPD-M20A IVDD system from Kyoto, Japan. The Macherey-Nagel precolumn (EC 4/3 Nucleodur 300-5 C4ec) and column (EC 250/3 Nucleodur 300-5 C4 ec) from Düren, Germany, were employed. The analysis was conducted at a temperature of 25 °C with a running time of 15 min, 1 mL/min flow rate and λ = 221 nm. ACN and 25.08 mM Na_2_HPO_4_ (pH 5.5) in 30:70 ratio was used as mobile phase.

#### ELISA

ELISA was conducted utilizing Sino Biological’s Human BMP-2 ELISA Kit (Art. No. KIT10426, Beijing, China) following the provided manufacturer’s guidelines. The kit included a 96-well plate coated with the capture antibody. After three washes with 300 µl wash buffer, 100 µl of each release sample was carefully dispensed into the wells. Additionally, a BMP-2 standard (0–2500 pg/mL) was prepared and treated similarly to the samples. Both the samples and the standard were completely aspirated within 15 min and left to incubate for 2 h at room temperature. Following three more washes, 100 µl of the detection antibody was added and allowed to incubate at room temperature for 1 h. Subsequently, the wells were washed three times, and 200 µl of the substrate solution was dispensed into each well. After a 20-minute incubation at room temperature in the dark, the color reaction was halted by adding 50 µl of the stop solution.

### Biocompatibility

All cell culture experiments were performed with MG-63 cells (ATCC-CRL 1429). In addition, the ceramics used for the cell culture experiments were sawn to a thickness of 2 mm and filled with ADA-gelatin as described before. The Live-Dead assay (PromoCell Live/Dead Cell Staining Kit II, Art. No. PK-CA70730002, Heidelberg, Germany), WST-1 assay (Roche Cell Proliferation Reagent WST-1, Art. No. 11644807001, Basel, Switzerland), and LDH assay (Roche Cytotoxicity Detection Kit (LDH), Art. No. 11644793001, Basel, Switzerland) were conducted in accordance with the manufacturer’s instructions. For the live-dead assay, 20,000 cells were used per sample, while 50,000 cells per sample were employed for both the WST-1 and LDH assays.

### Anti-microbial activity

To demonstrate that the antibiotics used were still microbially effective after 28 days, their minimum inhibitory concentration was determined. For this purpose, ISO Standard 20776-1 and EUCAST [40] were followed. Samples from release days 1, 2, 3, 6, 9, 14, 21, and 28 of daptomycin BMP-2 release were tested. The exact procedure is already described elsewhere [30, 38]. Defined dilutions of each concentration (original concentration as well as 4 above and 4 below the MIC) were prepared which were then tested for MIC in a microtiter experiment with *S. aureus* (ATCC 29213).

### Statistics

The data was expressed as mean ± standard deviation and subjected to analysis using one-way analysis of variance (ANOVA). The comparison of means was conducted using Fisher LSD. The level of statistical significance was established at *p* < 0.05. All calculations were carried out using OriginPro 2022 SR1 from OriginLabs in Northampton, MA, USA.

## Results

### Characterization of the microporous β-TCP

**Fig. 1:**
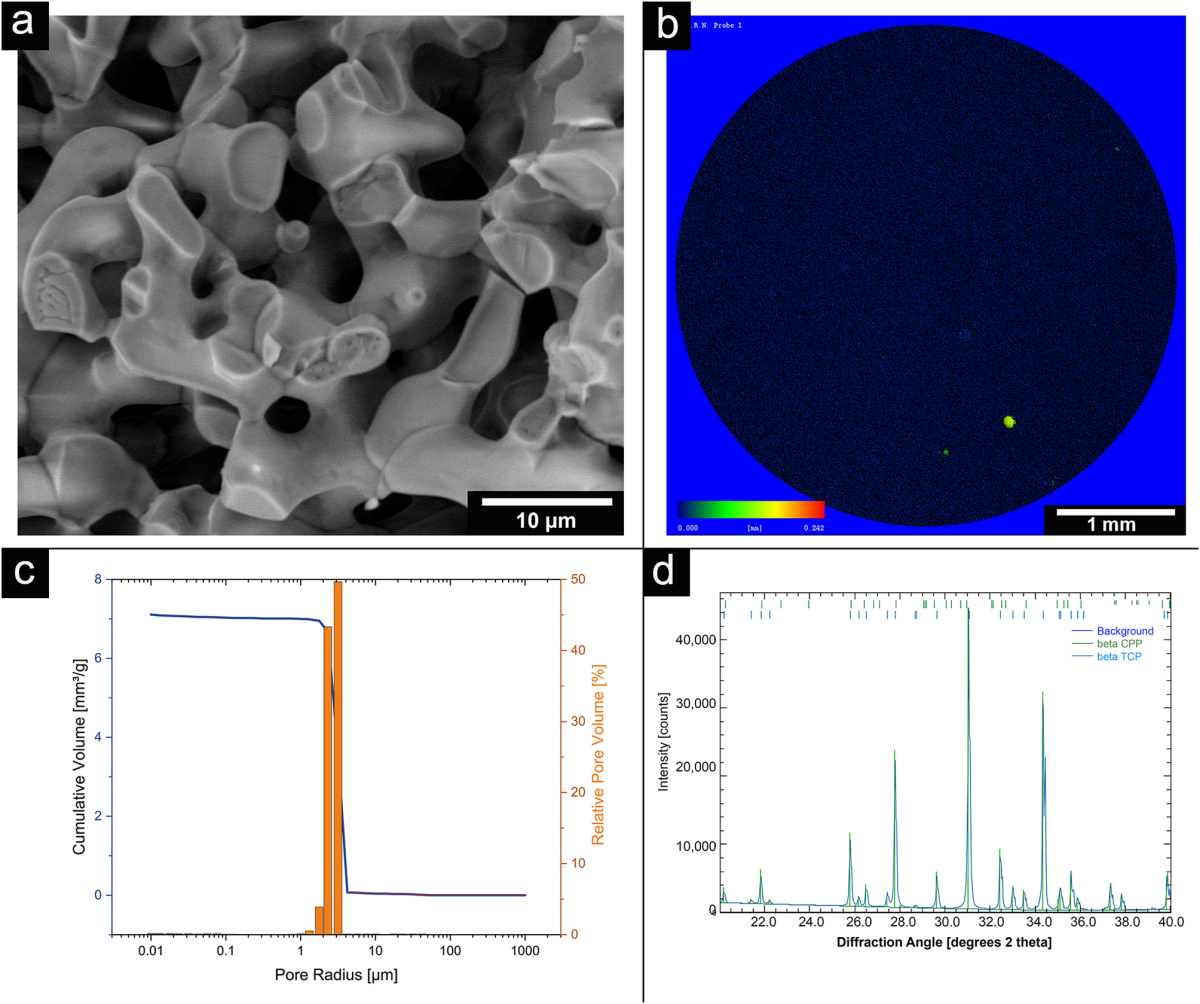
Overview of β-TCP characteristics: (**a**): ESEM Image HFW 46.6 μm (fracture surface); HV: 10 kV; LFD detector @ 100 Pa; (**b**): µCT of porosity of the β-TCP taken by Scanco Micro-CT 50 at 90 kV, 4 W, 44 µA at a resolution of 2 μm and an integration time of 5000 ms ; (**c**): Pore size distribution; measurements of the Pascal-140 porosimeter (1000 μm–1.4 μm; pressure increase to 0.1 kPa) in blue and the Pascal-440 porosimeter (1.4 μm–1.8 nm; pressure increase to 400 MPa) in purple (Porotec Pascal 140/440, Hofheim, Germany); (**d**): XRD pattern of the TCP (Bruker D8 Advance, Billerica, USA; Bragg-Brentano geometry; Cu anode; secondary graphite monochromator; scintillation counter; 40 kV/40 mA; 1°2-theta/ min; step size 0.02°2theta) and Rietveld Refinement with Profex 4.3

The mean pore size of the ceramics was determined to be 4.9 ± 0.4 μm and a total porosity of 45 ± 4% using scanning electron microscopy and porosimetry. The EDX shows a Ca/P ratio of 1.48, which identified the sample as β-TCP. The XRD measurement confirmed the result with the subsequent Rietveld refinement analysis (Profex 4.3., www.profex.org, Freeware). 99.5% β-TCP and traces of calciumpyprophpshate (CPP) from the manufacturing process could be detected. We came to similar conclusions in other studies of β-TCP [30, 32, 38] (pls. see Fig. 1).

### Characterization of the ADA-gelatin gel

**Fig. 2:**
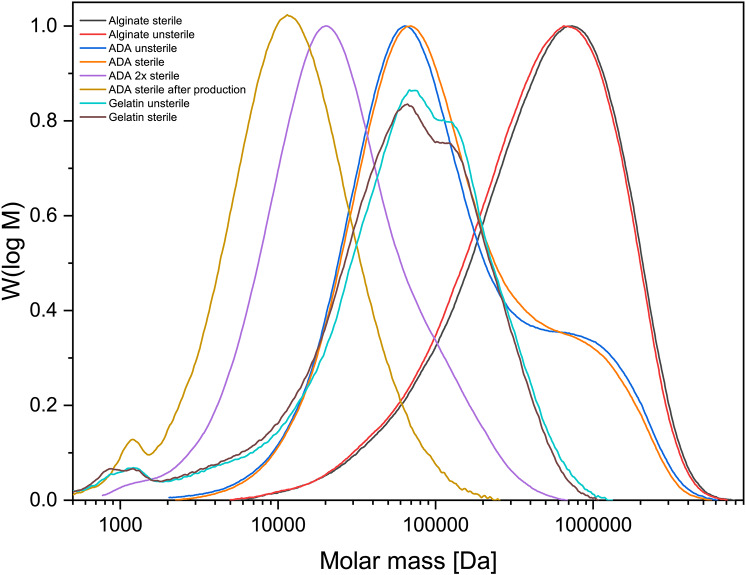
Molar mass distribution (unsterile vs. sterile) for Alginate, ADA and Gelatin

Both alginate and gelatin show no changes in molecular weight before and after plasma sterilization. Unsterile ADA as well as ADA sterile also show similar values Mn 52–55 kDa; Mw 298–320 kDa; Mz 1150–1300 kDa. The greatest differences can be observed in ADA that has been plasma sterilized after preparation. The values for Mn decrease from the range 50 kDa (before) to 7–13 kDa (after). PDI (Mw/Mn) halves from 5 to 6 to 2–3. The Table 1; Fig. 2 below shows an overview of the plasma-sterilized gels. It becomes clear that the influence of the sterilization process on the gelatin and alginate is not as great as on ADA.

**Table 1:** Molecular weights of alginate, ADA and gelatin at different times in the sterilization process; [*n* = 3]

Sample	M_*n*_ [kDa]	M_w_ [kDa]	M_z_ [kDa]	PDI (= M_w_/M_*n*_)
Alginate unsterile	198 ± 23	729 ± 89	1440 ± 99	3.68
Alginate sterile	201 ± 18	767 ± 57	1530 ± 187	3.81
ADA unsterile	52.4 ± 6,8	316 ± 26	1280 ± 111	6.03
ADA sterile	55.3 ± 5.3	298 ± 17	1160 ± 53	5.38
ADA sterile after manufacturing	6.91 ± 0.87	17.8 ± 2.1	39.8 ± 2.5	2.57
ADA 2x sterile	13.1 ± 1.4	41 ± 9	116 ± 12	3.13
Gelatin unsterile	16.3 ± 2.7	114 ± 9	254 ± 22	6.99
Gelatin sterile	15.9 ± 3.3	105 ± 8	231 ± 16	6.61

The complex viscosity of the ADA was found to be 0.2 ± 0.02 Pa∙s independent of the frequency. The shear viscosity of gelatin, on the other hand, was very strongly dependent on the frequency (3 Pa∙s at 1 Hz, 50 Pa∙s at 0.1 Hz and 0.3 Pa∙s at 10 Hz).

### Release kinetics of dual release of daptomycin and BMP-2

The release of daptomycin was recorded over the entire measurement period of 28 days. This resulted in a burst release of 71% or a concentration of 1026.05 ± 84.28 µg/ml. On the last day, a concentration of 0.18 ± 0.67 µg/ml was still detectable. The complete release is summarized in Table 2. In contrast, the burst release of BMP-2 was only 4.8 ± 7.1% or 58.8 ± 90 ng/ml. BMP-2 was also detected throughout the 28-day study period, but this time by ELISA. The summary of the detected DAP and BMP-2 concentrations is shown in Table 2. According to Diederen et al. [41] the daptomycin concentration lies within the MIC (0.125–1 mg/l) against *S. aureus*. Based on the initial weight during production, 105.13 ± 9.14% daptomycin was released, i.e. the entire initial weight, but only 0.72 ± 0.16% BMP-2.

**Table 2:** Overview of released daptomycin and BMP-2 concentrations

Release period [d]	Daptomycin-Release [µg/mL]	BMP-2 Release [ng/mL]
1	1026.05 ± 84.28	59.80 ± 90.0
2	318.01 ± 34.85	101.86 ± 92.04
3	72.43 ± 48.64	669.00 ± 148.40
6	8.62 ± 2.65	208.96 ± 43.20
9	2.76 ± 0.18	71.54 ± 47.63
14	1.45 ± 1.30	148.31 ± 100.86
21	0.37 ± 0.98	1.75 ± 6.14
28	0.18 ± 0.67	2.63 ± 9.15
**Recovery [%]**:	105.13 ± 9.14	0.72 ± 0.16

**Fig. 3:**
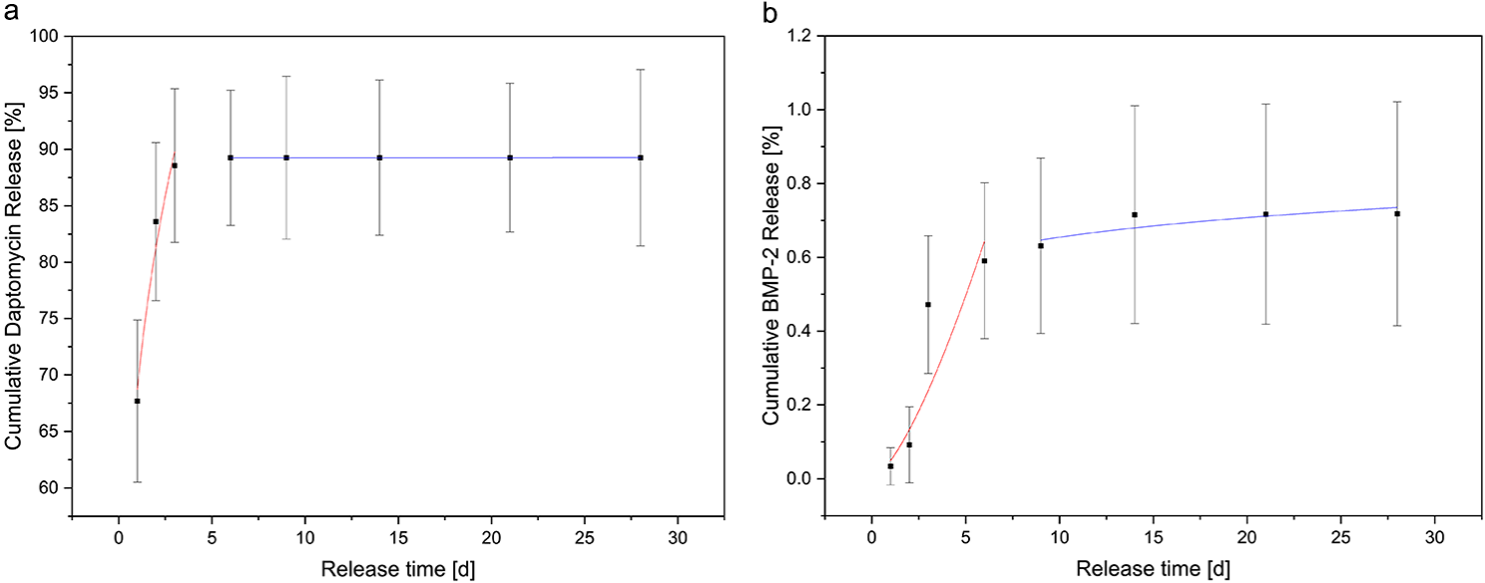
Fitting of release according to Ritger/Peppas at the beginning in red and at the ending in blue. Cumulative daptomycin release on the left and cumulative BMP-2 release on the right

The analysis of the release kinetics according to Ritger-Peppas showed low values for n for daptomycin (see Table 3). This corresponded to anomalous release kinetics. In order to carry out a meaningful fitting for the BMP-2 release, the values on day 0 and 2 were not taken into account. There was also an anomalous release of BMP-2, but with significantly higher values of the diffusion exponent at the beginning than with daptomycin (Fig. 3). The beginning and the end of the release were considered separately, as the analysis according to Ritger-Peppas had the highest significance in the first 20% of the release.

**Table 3:** Overview of used parameters for fitting according to Ritger/Peppas (y = k ∙ x^n^), fitting at the beginning labelled with start and at the ending with end, R: Pearson correlation coefficient

Daptomycin		BMP-2	
n_start_	0.11	n_start_	0.87
k_start_	75.14	k_start_	0.11
R^2^_start_	0.54	R^2^_start_	0.77
n_end_	0.00004	n_end_	0.10
k_end_	89.41	k_end_	0.52
R^2^_end_	0.37	R^2^_end_	0.57

### Biocompatibility

**Fig. 4:**
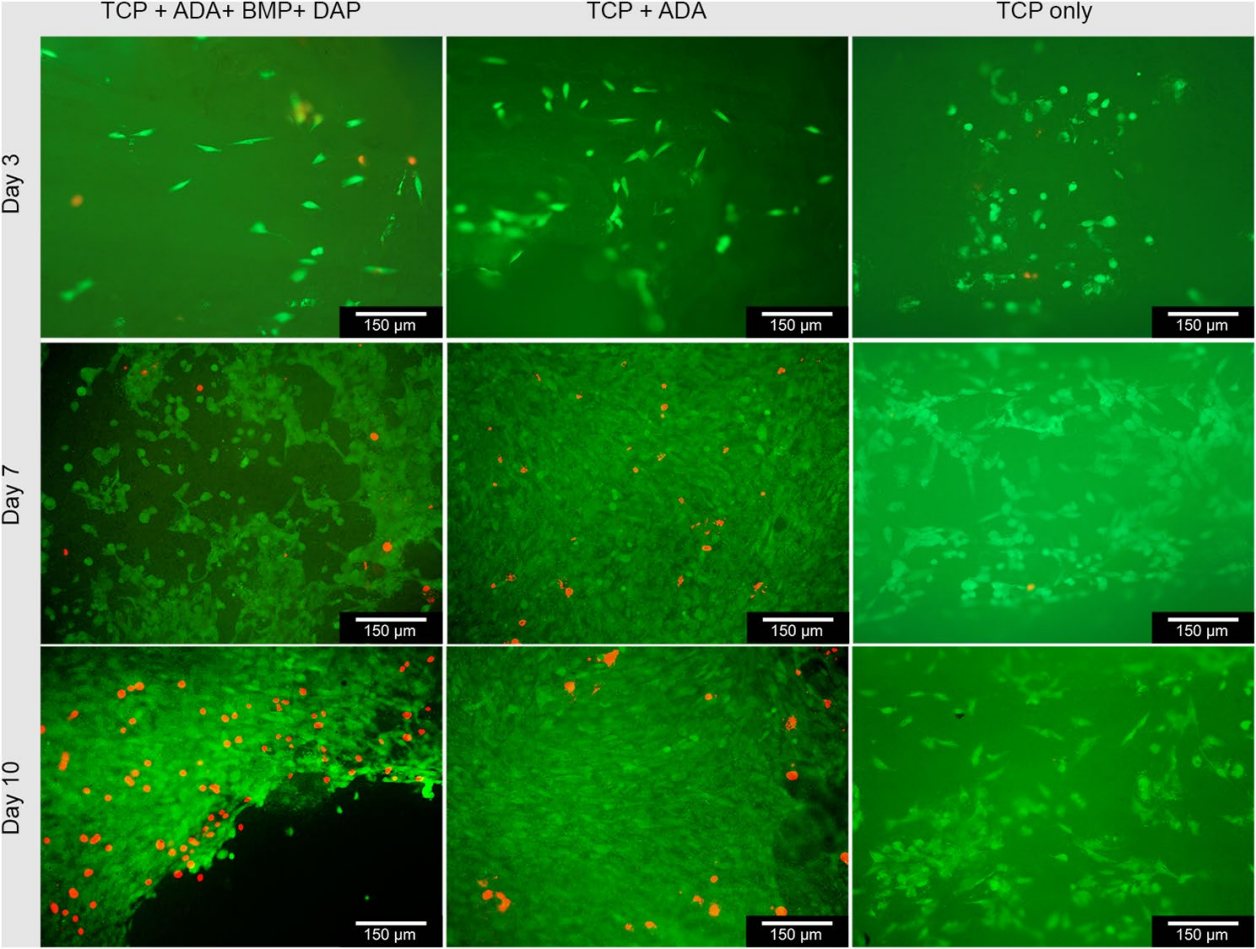
Exemplary live dead staining’s of MG-63 cells on β-TCP filled with ADA/BMP/DAP; ADA and empty TCP after 3, 7 and 10 days; living cells in green, dead cells in red, images taken with Olympus BX-53 Fluorescence microscope; 10× magnification

In live/dead staining, living cells predominated with 65–78% on days 3, 7 and 10. The empty ceramic as control showed 89–96%, whereas ceramics with ADA/gelatin (control 2) showed 82–97% live cells. An overview of the results is shown in Table 4. We introduced the intermediate section because there were cells that were stained both green and red and were clearly not dead in the microscopy. Exemplary images of the live dead staining are shown in Fig. 4.

**Table 4:** Relative cell counts MG-63 cells on composite vs. empty ceramics

Sample	Relative cell count [%]		
	Alive	Intermediate	Dead
**Day 3**			
Dap/BMP-2	74.85 ± 48.16	5.56 ± 2.82	19.59 ± 9.08
Control (empty ceramics)	95.93 ± 14.21	0 ± 0	4.07 ± 1.20
Control 2 (ADA/Gel no drugs)	96.48 ± 31,22	0 ± 0	3.52 ± 1.93
**Day 7**			
Dap/BMP-2	65.83 ± 27.14	0 ± 0	34.17 ± 23.41
Control (empty ceramics)	93.53 ± 11.33	0 ± 0	6.47 ± 1.48
Control 2 (ADA/Gel no drugs)	82.95 ± 31.48	0 ± 0	17.05 ± 4.80
**Day 10**			
Dap/BMP-2	77.41 ± 63.13	0 ± 0	22.59 ± 22.97
Control (empty ceramics)	89.07 ± 53.61	0 ± 0	10.93 ± 3.84
Control 2 (ADA/Gel no drugs)	82.79 ± 27.79	0 ± 0	17.21 ± 10.67

Intermediate = cells that were stained both green and red and were clearly not dead in the microscopy

**Fig. 5:**
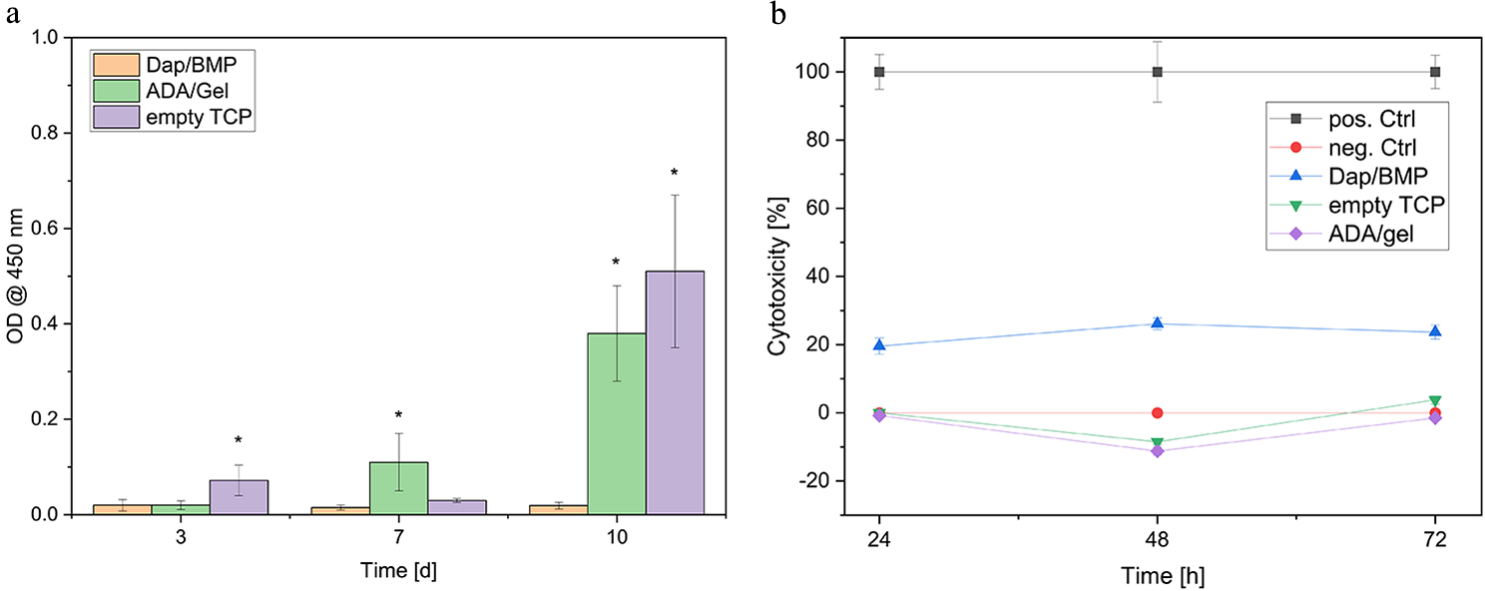
Overview cell viability (**a**) and cytotoxicity (**b**). (*): Result was statistically significantly different from the others of the same day with *p* < 0.05

Cell proliferation (WST-I) of MG-63 cells (see Fig. 5a) on the composites of ADA-gelatin, daptomycin and BMP-2 was lower compared to the controls (empty TCP) and TCP with ADA-gelatin. In addition, proliferation was approximately constant throughout the study period, while it increased in both the empty and ADA/gelatin-filled ceramics. Regarding cytotoxicity (LDH) in Fig. 5b, both controls (empty TCP, gel-filled TCP) showed similar values to the negative control (cells only) in the range of 0% (negative values also correspond to 0%) over the 3-day study period. The combination of daptomycin and BMP-2 showed almost constant values of 19–22%. There was no statistically significant difference between ADA/gel, empty TCP and the negative control.

### Antimicrobial activity

The MIC for daptomycin BMP-2 release ranged from 0.475 to 1 mg/l (see Table 5). For release days 14 to 28, no antimicrobial activity was detected. The inoculum control (GC) provided a bacterial concentration of 7 × 10^5^ mL^-1^.

**Table 5:** Overview of the antimicrobial activity of the released Daptomycin exemplary on one sample

Sample	Dilution levels of the original concentration [mg/l]												
	16	8	4	2	1	0.5	0.25	0.125	0.063	0.031	GC	EW	MIC
Control	–	–	–	–	–	–	+++	+++	+++	+++	+++	–	0.5
	1291	8	4	2	1	0.5	0.25	0.125	0.063	0.031	GC	EW	MIC
D1P1	–	–	–	–	–	–	+++	+++	+++	+++	+++	–	0.5
	308	8	4	2	1	0.5	0.25	0.125	0.063	0.031	GC	EW	MIC
D2P1	–	–	–	–	–	–	+++	+++	+++	+++	+++	–	0.5
	110.5	8	4	2	1	0.5	0.25	0.125	0.063	0.031	GC	EW	MIC
DT3P1	–	–	–	–	–	+++	+++	+++	+++	+++	+++	–	1
	18.59	8	4	2	1	0.5	0.25	0.125	0.063	0.031	GC	EW	MIC
D6P1	–	–	–	–	–	–	+++	+++	+++	+++	+++	–	0.5
	–	0.95	0.475	0.238	0.119	0.059	0.03	0.015	0.007	0.004	GC	EW	MIC
D9P1	/	–	–	+++	+++	+++	+++	+++	+++	+++	+++	–	0.475
	–	0	0	0	0	0	0	0	0	0	GC	EW	MIC
D14P1	/	++	+++	+++	+++	+++	+++	+++	+++	+++	+++	–	> 0
	–	0	0	0	0	0	0	0	0	0	GC	EW	MIC
D21P1	/	++	+++	+++	+++	+++	+++	+++	+++	+++	+++	–	> 0
	–	0	0	0	0	0	0	0	0	0	GC	EW	MIC
D28P1	/	++	+++	+++	+++	+++	+++	+++	+++	+++	+++	–	> 0

D1 through D28 represents day 1 through 28; GC = growth control, only bacteria; EW = empty well; (-) no growth; (+) growth; (++) strong growth; (+++) very strong growth

## Discussion

Both EDX and XRD showed that the RMS ceramic consisted of β-TCP. Rietveld refinement analysis also confirmed this. In ESEM, µCT and porosimetry the pore structure could be verified (requirement ca. 5 μm pore diameter). These findings were in agreement with existing work on the ceramic. Rietveld refinement analysis also confirmed this [30, 32, 42]. Previous works have also successfully shown that the ceramic is very well integrated into the bone and degrades completely over time [34, 43]. GPC measured a molar mass of 198 kDa for alginate and 52.4 kDa for ADA. Sarker et al. [44] determined the molar masses of alginate and ADA via their viscosities. This resulted in 422.3 ± 5.3 kDa for alginate and 185.5 ± 2.8 kDa for ADA, significantly lower molar masses than those determined via GPC in this work. This again showed the wide variation in the batches of alginate used, although the same alginate with the same order number from Sigma was used in both papers. In loading experiments by Seidenstücker et al. [30] performed with similar procedures, alginate with a molar mass Mn of 93 ± 18 kDa determined in GPC was used; before plasma sterilization, this value was 343 ± 45 kDa. Again, the molar mass of the alginate was higher than that of the alginate used in this work. It is worth noting that in the study by Seidenstuecker et al. [30], the molar mass of alginate underwent a significant reduction with plasma sterilization, whereas in our current investigation, plasma sterilization had minimal impact on the molar mass of alginate. This highlights that low-temperature hydrogen peroxide gas/plasma sterilization proves to be a gentler process. In a comparable loading procedure described in the literature, a 2.5% alginate gel required approximately 10 ± 3.1 min for complete loading of the ceramics [28]. Rheological analysis revealed a complex viscosity of 0.35 Pa∙s at a frequency of 10 rad/s (= 1.6 Hz). In our present work, the complex viscosity of the ADA gelatin gel at the same frequency increased from 0.09 Pa∙s to 0.16 Pa∙s after 10 min and reached 0.5 Pa∙s after 30 min during an exemplary crosslinking (measurement 1). Despite slight differences in measurement parameters, this resulted in a comparable expected loading time for the ADA gelatin gel. In comparison with another study [45], where both the gel preparation and rheological measurement parameters aligned, a significantly longer crosslinking time was observed. In Sarker et al. [45], this time was reported as 8.2 min, while in our current work, it was at least 43 min. The burst release of daptomycin at 71% is significantly greater than comparable results for vancomycin from alginate in a previous work [30]. There, the burst release was only 35.2 ± 1.5%. In contrast to the present work, however, alginate and not ADA gelatin was used. The release of BMP-2 was significantly lower than in previous studies, remaining in the single-digit percentage range. In contrast, in Kissling’s study [29], 45.4% was released within the first 48 h. The percentage of live cells on daptomycin and BMP-2-containing hydrogel was between 10 and 20% lower than on the blank ceramic. In comparison to this, the ADA-gelatin loaded ceramic showed a lower percentage of live cells of 5–20%. Nevertheless, the cytotoxicity measurements showed little difference from other work with CDHA [46]. The differences are due to the effects of BMP-2 concentration, as also seen in cell proliferation [29]. However, there is a habituation effect after 1 week which is expressed by the fact that an increasing number of living cells could be observed. This contrasts with the observations of the antimicrobial efficacy of daptomycin against S. aureus. The efficacy was only 9 days, although the concentrations were still within the range described by Diederen et al. [41]. EUCAST even demand a significantly higher classification of the MIC with 1 mg/mL [40]. Hall et al. [47] described a similar release behavior of daptomycin. They were able to determine a release from PMMA beads over 86.7 ± 7.6 h depending on the initial concentration. However, the burst release also increased linearly with the higher initial concentration and a comparable burst release of 42% was determined as in the present work. Unfortunately, antimicrobial investigations were not carried out by Hall et al. [47]. In the work of Silva at al [48], a release from chitosan nanoparticles was described. However, the release was already completed within 3 h. Nevertheless, they were able to determine a similar MIC between 0.5 and 1.0 mg/mL as we did. However, no conformational changes were observed by us on the daptomycin by means of a shift in the mean transit time or by peak changes, which would indicate a degradation of the daptomycin and a less antimicrobial activity (in presence of BMP-2). This work focussed on the treatment of osteomyelitis caused by Gram-positive bacteria, in particular Staphylococcus aureus as a well-established standard pathogen. In future, antibiotics against gram-negative bacteria should also be tested in the composite. A translation of the composite into clinical application could lead to its use as a dowel containing active ingredients in cruciate ligament replacements in the knee joint to prevent infection. Intraoperative cutting by the surgeon with individualised loading or preoperative production using CT data is also conceivable. The concurrent deployment of two distinct compounds, Daptomycin (an antibiotic) and BMP-2 (a bone morphogenetic protein), could give rise to intricate interactions. Predicting and managing the synergistic outcomes between these agents may pose challenges, possibly resulting in unforeseen repercussions. It is imperative to strike a delicate equilibrium in the release rates of Daptomycin and BMP-2. Imbalances in either substance’s dosage could impact the treatment’s overall efficacy. Determining the ideal release proportions may necessitate meticulous calibration and extensive testing.

## Conclusion

The dual release of daptomaycin and BMP-2 occurred over 28 days from the composite of microporous ceramic and ADA gelatin gel developed by us as the active carrier. The antimicrobial activity was also 9 days. Compared with previous release tests using nanoparticles (in the hour range) or PMMA (3 days), the new method is clearly superior despite the reduced antimicrobial activity. Alginate is not completely biodegradable by the human body. A hydrogel that could be completely degraded enzymatically by the human body would be even more suitable for controlled release of active ingredients.

## Data Availability

The data presented in this study are available on request from the corresponding author.
